# Short-interval intracortical inhibition: Comparison between conventional and threshold-tracking techniques

**DOI:** 10.1016/j.brs.2018.03.002

**Published:** 2018

**Authors:** Gintaute Samusyte, Hugh Bostock, John Rothwell, Martin Koltzenburg

**Affiliations:** a Sobell Department of Motor Neuroscience and Movement Disorders, UCL Institute of Neurology, Queen Square, WC1N 3BG, London, United Kingdom; b Department of Clinical Neurophysiology, National Hospital for Neurology and Neurosurgery, UCLH NHS Foundation Trust, Queen Square, WC1N 3BG, London, United Kingdom

**Keywords:** Threshold-tracking TMS, Short-interval intracortical inhibition, Reliability, Intraclass correlation coefficient, Coefficient of repeatability, A-SICI, short-interval intracortical inhibition obtained by conventional paradigm, CR, coefficient of repeatability, EMG, electromyography, ICC, intraclass correlation coefficient, IQR, interquartile range, MEP, motor evoked potential, rmANOVA, repeated measures analysis of variance, T-SICI, short-interval intracortical inhibition obtained by threshold-tracking, AT, A-SICI - T-SICI recording protocol sequence, TA, T-SICI - A-SICI recording protocol sequence, TS_1mV_, test stimulus intensity to evoke a peak-to-peak MEP of 1 mV

## Abstract

**Background:**

Short-interval intracortical inhibition (SICI) is conventionally measured as the *relative amplitude reduction* of motor evoked potentials (MEPs) by subthreshold conditioning stimuli. In threshold-tracking SICI (T-SICI), stimulus intensity is instead adjusted repeatedly to maintain a constant MEP and inhibition is measured as the *relative threshold increase*. T-SICI is emerging as a useful diagnostic test, but its relationship to conventional amplitude SICI (A-SICI) is unclear.

**Objective:**

To compare T-SICI and its reliability with conventional A-SICI measurements.

**Methods:**

In twelve healthy volunteers (6 men, median age 30 years), conventional and T-SICI were recorded at conditioning stimuli (CS) of 50–80% resting motor threshold (RMT) and interstimulus interval of 2.5 ms. Measurements were repeated on the same day and at least a week later by a single operator.

**Results:**

Across the CS range, mean group T-SICI showed a strong linear relationship to the mean group values measured by conventional technique (y = 29.7–0.3x, R^2^ = 0.99), but there was considerable interindividual variability. At CS 60–80% RMT, T-SICI had excellent intraday (intraclass correlation coefficient, ICC, 0.81–0.92) and adequate-to-excellent interday (ICC 0.61–0.88) reproducibility. Conventional SICI took longer to complete (median of 5.8 vs 3.8 min, p < 0.001) and tended to have poorer reproducibility (ICC 0.17–0.42 intraday, 0.37–0.51 interday). With T-SICI, smaller sample sizes were calculated for equally powered interventional studies.

**Conclusion:**

The close relationship between conventional and T-SICI suggests that both techniques reflect similar cortical inhibitory mechanisms. Threshold-tracking measurements of SICI may be able to improve reproducibility, to shorten acquisition time and to reduce sample sizes for interventional studies compared with the conventional technique.

## Highlights

• Threshold-tracking SICI correlates well with conventional measures.
• It shows a trend towards better reproducibility on a group level.
• Threshold-tracking SICI is potentially quicker to obtain.
• It may require a smaller sample size in interventional studies.

## Introduction

Transcranial magnetic stimulation (TMS) is a non-invasive method that can be employed to study inhibitory and excitatory microcircuits of the brain [1]. Short-interval intracortical inhibition (SICI) is one of the most widely studied inhibitory phenomena. When subthreshold conditioning and subsequent suprathreshold test stimuli are delivered through the same coil at interstimulus intervals (ISIs) of 1–6 ms, their interaction results in suppression of the motor evoked potential (MEP) amplitude [2]. Two distinct phases of SICI have been observed at 1 ms and 2.5 ms ISIs [3,4]. While the mechanism of the first phase is not fully understood, SICI at an ISI of 2.5 ms is thought to reflect gamma-aminobutyric acid (GABA) mediated inhibition in the motor cortex [5].

Conventional TMS paradigms for SICI use a *constant stimulus* approach in which fixed intensity stimuli are applied and multiple responses are averaged to obtain a reliable estimate [2]. SICI is then expressed as the reduction of the average conditioned MEP amplitude in comparison to the average control MEP size. Due to high trial-to-trial variability of MEPs, it is recommended to obtain at least 8–10 responses for each condition [5]. If multiple conditions are investigated, recordings may become time-consuming. Another potential disadvantage of this approach is that it assumes that the resting motor threshold (RMT) remains constant throughout the lengthy recording. However, it may change considerably due to biological or technical factors [5,6]. Thus, the pre-defined conditioning stimulus (CS) intensity, commonly set as a percentage of RMT, may become suboptimal for eliciting SICI.

By contrast, a *constant response* approach is used in threshold-tracking. This method was pioneered by Bostock and colleagues [3,7]. Its main principle is that the stimulation intensity is dynamically adjusted to maintain the response at a predetermined target level. Therefore, if the CS suppresses the response, the test stimulus intensity will increase to counteract this effect. In this technique, RMT is the control condition and SICI is reflected by the relative increase in test stimulus intensity over RMT (the bigger the increase, the stronger the inhibition). The main advantage of this paradigm is that any drifts in motor threshold are continuously monitored and adjusted for.

Impairment of SICI has been reported across a wide range of neurological disorders [[8], [9], [10], [11], [12], [13], [14], [15], [16], [17], [18]], but due to its large variability between patients and overlap with normal subjects, conventional SICI has limited clinical diagnostic use [19,20]. However, T-SICI is emerging as a potentially useful diagnostic test [[21], [22], [23], [24]]. Recent data shows its diagnostic utility in distinguishing amyotrophic lateral sclerosis from mimic disorders [21] and as a possible biomarker for the effect of therapeutic interventions [25].

While reliability of conventional SICI measurements has been previously studied [8,[26], [27], [28], [29]], little is known about reliability of threshold-tracking TMS and its comparability with conventional technique. Therefore, the aim of this study was a head-to-head comparison of the two techniques for SICI measurement. We tested the hypothesis that threshold-tracking paradigms which allow monitoring of the naturally occurring fluctuations in RMT can improve the reliability of SICI making it a preferred tool for both clinical practice and research.

## Methods

The study was carried out in accordance with the Declaration of Helsinki, approved by local ethics committee, and a written informed consent was obtained from participants prior to investigations.

### Subjects

16 healthy volunteers with no known neurological disorder or contraindications for TMS and not on any regular medication were recruited for the study. Twelve subjects (6 men; median age 30 years, age range 23–52 years) completed the full set of experiments. Four subjects were excluded due to inability to maintain relaxation of the hand (n = 1) or incomplete stimulation sessions due to coil overheating (n = 3).

### Experimental setup

During the experiment participants were comfortably seated in an armchair and instructed to stay relaxed but alert. Surface electromyography (EMG) was recorded from the relaxed right first dorsal interosseous muscle with Ag/AgCl electrodes (Kendall 5500 Diagnostic Tab Electrodes, Covidien, Dublin, Ireland) placed in a belly-tendon montage. The EMG signal was amplified (×600 gain), filtered (10–3000 Hz), and sampled at 10 kHz using the EA-2 amplifier of a Viking Select EMG Unit (Nicolet Biomedical Inc, Madison, WI, USA). EMG of the target muscle was displayed on a screen in front of the subjects as a visual feedback to aid maintaining relaxation of the hand.

TMS was carried out using two Magstim 200^2^ stimulators connected in BiStim mode and a figure-of-eight D70^2^ coil (Magstim, Whitland, UK). Stimulus delivery and data acquisition were controlled by QTRACW software (©Institute of Neurology, University College London, London, UK, distributed by Digitimer Ltd. at www.digitimer.com) using bespoke recording protocols.

The coil was hand-held over the left hemisphere with the handle pointing postero-laterally at a 45° angle to the mid-sagittal line to induce posterior-to-anterior flow of the current in the motor cortex. Magnetic stimuli were delivered at 4.1 s intervals.

Once the hotspot was identified, an automated stimulation protocol was started, allowing a single operator to carry out the whole recording without the need to reposition the coil or manually control the stimulator.

### Resting motor threshold

In conventional protocols, RMT is usually defined as the minimal stimulus intensity required to obtain a peak-to-peak MEP amplitude of >0.05 mV in 50% of consecutive trials and 10 out of 20 trials are recommended for obtaining reliable results [5]. In threshold-tracking paradigms, RMT is defined as the stimulation intensity required to maintain the target MEP which is usually set as peak-to-peak amplitude of 0.2 mV (further referred to as RMT_0.2mV_) [3,30]. We used threshold-tracking to obtain RMT_0.2mV_ estimates employing a proportional tracking mode in which stimulus intensity is adjusted proportionally to the percentage error in the logarithm of the previous response [3] with the maximum stimulus step limited to 2% MSO. Tracking was deemed stable when the MEP hit or oscillated around the target amplitude six times. RMT_0.2mV_ was then used to set CS intensities for both conventional and T-SICI measurements to allow direct comparison between the techniques.

### Short-interval intracortical inhibition

SICI measurements at an ISI of 2.5 ms and CS intensities of 50%, 60%, 70%, and 80% RMT_0.2mV_ were obtained using both conventional (‘amplitude’, A-SICI) and threshold-tracking (T-SICI) techniques. ISI of 2.5 ms was chosen as SICI at this interval is thought to reflect GABA Aα2,3 receptor mediated inhibition [31] and can potentially serve as a biomarker of the effect of GABA A receptor modulating drugs. As the relationship between SICI and CS intensity is non-linear and varies between individuals [5,32], a range of CS intensities was used to explore whether SICI recruitment curve may provide a more reliable measure than SICI estimates at a single CS intensity.

For A-SICI, test stimulus intensity required to evoke MEPs of peak-to-peak amplitude of approximately 1 mV (TS_1mV_) was determined by threshold tracking (target set at 1 mV). Test and conditioning stimuli of fixed intensity were then used to record fifteen MEPs for control and each SICI condition in a pseudorandom order.

**Fig. 1:**
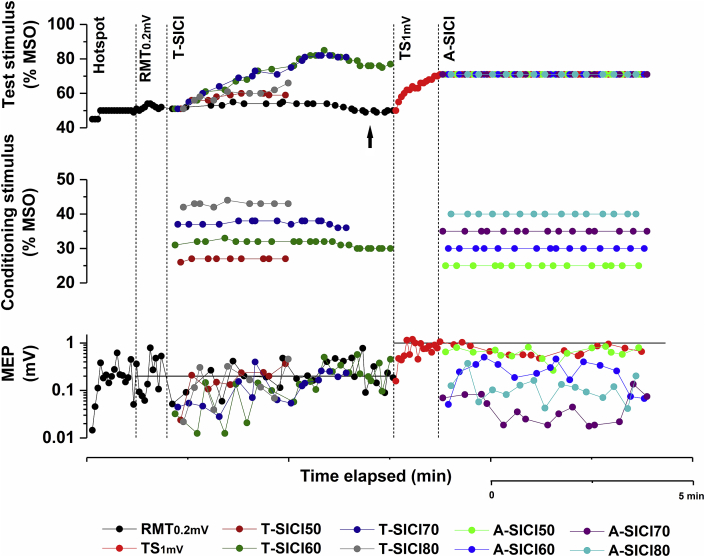
**Representative illustration of the automated stimulation protocol in a single subject.** After finding the hotspot, the following parameters were recorded (separated by vertical dotted lines): resting motor threshold (RMT_0.2mV_); SICI using constant-response (T-SICI) and constant-stimulus (A-SICI) techniques at conditioning stimulus intensities of 50–80% RMT_0.2mV_. The target response of 1 mV (TS_1mV_) was determined by tracking to this target and this value was then used for the entire A-SICI protocol. Test stimulus intensity (top), conditioning stimulus intensity (middle), and motor evoked potential (MEP) amplitude (bottom) were recorded throughout the protocol for each condition (indicated by different colours). All stimulation intensities were adjusted automatically by the QTRACW software, thus enabling a single operator to carry out the whole recording without having to reposition the TMS coil or to manually adjust the intensity of the stimuli. Horizontal solid lines (bottom graph) represent target MEP size: 0.2 mV for RMT_0.2mV_ and T-SICI, 1 mV for TS_1 mV_ and A-SICI. Note that RMT_0.2mV_ drifts in T-SICI part (indicated by arrow) and conditioning stimulus intensities are adjusted accordingly, whereas similar compensations are not possible in A-SICI part (conditioning stimulus intensity for A-SICI was set based on one RMT_0.2mV_ estimate). (For interpretation of the references to colour in this figure legend, the reader is referred to the Web version of this article.)

For T-SICI, RMT_0.2mV_ was tracked throughout and CS intensities were adjusted depending on the fluctuations in RMT_0.2mV_ to maintain them as a constant fraction of RMT_0.2mV_. Paired and control (RMT_0.2mV_) stimuli were delivered in a pseudorandom order. Tracking was started at RMT_0.2mV_ intensity and proportional tracking mode was used. Test stimulus intensities were adjusted to maintain the target response of 0.2 mV when preceded by CS. Tracking for each SICI condition was stopped when the conditioned MEP hit or oscillated around the target six times. A representative individual recording of the experiment is shown in Fig. 1.

SICI analysis was performed offline using QTRAC-P software (part of QTRACW package). For A-SICI, the peak-to-peak MEP amplitudes were averaged for each condition and SICI was expressed as [conditioned MEP/test MEPx100%] [2], with values below 100% reflecting inhibition. For T-SICI, stimulation intensities (y axis) were plotted against logarithmically transformed MEP amplitudes (x axis) and a linear least squares model was fitted, excluding MEP values outside 0.02–2 mV range. This was done based on previous observations which showed that relationship between logarithmically transformed MEP amplitudes and magnetic stimulus intensity was approximately linear in this range [3]. The y value at the intercept of a linear regression line with the target (0.2 mV) was defined as the threshold. T-SICI was expressed as [(conditioned threshold–RMT_0.2mV_)/RMT_0.2mV_x100%] [30], with positive and negative values indicating inhibition and facilitation, respectively.

SICI at individual CS intensities, combined measure of SICI slope (sum of SICI at all CS intensities) and peak SICI (maximum inhibition irrespective of CS intensity) were used for the analysis.

### Experiment design

A-SICI and T-SICI were obtained at the same time of day in immediate succession over the same motor hotspot using a stimulation protocol incorporating both techniques (Fig. 1). To control for period effects, two stimulation sequences were employed: 1) T-SICI, followed by A-SICI (TA), and 2) A-SICI, followed by T-SICI (AT). Subjects were pseudorandomly assigned to one of these sequences for the whole experiment (six per protocol). SICI measurements were made on two experimental days separated by at least one week during a similar time of day. On each day, the stimulation protocol was applied twice using the same sequence with a short 10-min break. Between these sessions, the TMS coil was replaced to prevent overheating and hotspot identified anew. In three subjects with high RMT_0.2mV_ (>60% MSO), the full stimulation protocol could not be completed as the coil overheated during the second portion of the sequence (T-SICI in one, A-SICI in two subjects), thus they were excluded from the study. All measurements were carried out by a single operator.

### Statistical analysis

Statistical analysis was performed using IBM SPSS Statistics Version 22.0 (IBM Corp., Armonk, NY, USA). Data was checked for normality using Shapiro-Wilk test. For normally distributed data (Shapiro-Wilk test, p > 0.05) parametric tests were used for comparisons between groups and repeated measurements. Non-parametric tests (Wilcoxon signed rank test, Mann-Whitney *U* test, Friedman test) were used for non-normally distributed data.

Repeated measures analysis of variance (rmANOVA) was used to compare TMS parameters between the sessions (4 levels). If assumption of sphericity was violated (Mauchley's test of sphericity, p > 0.05), Greenhaus-Geisser corrections were applied. If significant main effects were identified, *post hoc* pairwise comparisons with Bonferroni adjustment were performed.

Pearson's correlation coefficient (r) was used to determine association between A-SICI and T-SICI. Intraclass correlation coefficient (ICC) was used to assess reproducibility of TMS measurements. Two-way random model, absolute agreement type, single measures [ICC (2,1)] and averaged measures [ICC (2,k)] were used [33] with the following categories of reliability: excellent – ICC>0.75; intermediate-to-good – 0.4 ≤ ICC≤0.75; poor – ICC<0.4 [34]. Cohen's kappa was used to assess the agreement between the CS intensity at which peak inhibition was observed within and between the experimental days.

Bland–Altman plots were constructed to assess the repeatability of SICI measurements [35]. Coefficient of repeatability (CR) was calculated using formula:

CR = 1.96 × *SD*_*WS*_ x √2 = 2.77 × *SD*_*WS*_ [36,37]

The within-subject standard deviation (SD_WS_) was obtained by taking a square root of within-subject variance partitioned by fitting one-way ANOVA model with Subject as a factor [37]. SD_WS_ reflects the standard error of measurement (SEM_eas_), which defines the accuracy of a measure (i.e. size of measurement error) irrespective of between-subject variability [8].

Data is presented as mean ± standard deviation (SD) when normally distributed or as median and interquartile range (IQR) of the 25th and 75th percentile, if non-normally distributed.

## Results

### TMS parameters

**Fig. 2:**
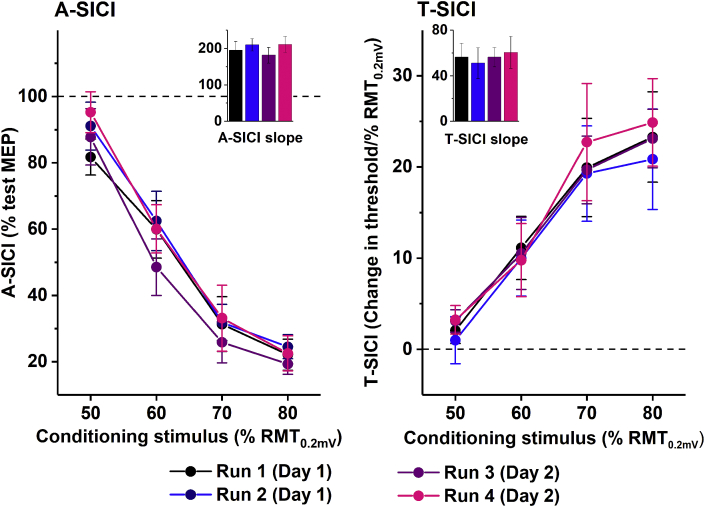
**SICI recruitment curve.** No significant difference was observed between the sessions in both mean group A-SICI and T-SICI, neither at any single conditioning stimulus intensity, nor at peak or combined slope (inset) measurements (rmANOVA, F = 0.21–0.76, p > 0.5; Friedman test, p > 0.3). Dotted lines indicate the test condition (test MEP for A-SICI, RMT_0.2mV_ for T-SICI), error bars represent standard error of the mean.

A summary of TMS parameters is presented in Table 1. The interval between two recording days ranged from 1 to 14 weeks (median of 2 (IQR 2) weeks). Across four sessions, all measurements were normally distributed (Shapiro-Wilk test, p > 0.05), except for T-SICI60. Test stimulus intensity to produce a peak-to-peak MEP of 1 mV (TS_1mV_) was 118.4 ± 10.6% RMT_0.2mV_. No significant difference was observed between the sessions in mean group RMT_0.2mV_ and TS_1mV_ (rmANOVA, F_1.7,18.2_ = 1.375, p = 0.274 and F_3,33_ = 0.506, p = 0.681, respectively) or mean group A-SICI and T-SICI, neither at individual CS intensities, nor peak or slope measurement (Fig. 2).

**Table 1:** Reliability of corticospinal excitability parameters.

TMS parameter	Mean ± SD^a^	Intraclass correlation coefficient						Coefficient of repeatability (SDC)		
		Intraday		Interday		Interday averaged^b^		Intraday	Interday	Interday averaged^b^
		ICC(2,1)^d^	ICC (2,k)^e^	ICC (2,1)^d^	ICC (2,k)^e^	ICC (2,1)^d^	ICC (2,k)^e^			
RMT_0.2mV_	48.6 ± 6.7	0.935	0.966	0.678	0.808	0.811	0.895	5.5	11	8.5
TS_1mV_	57.6 ± 9.4	0.915	0.955	0.725	0.841	0.750	0.857	10	14	15
A-SICI50	89 ± 14.2	0.123	0.218	0.144	0.252	0.286	0.444	58	62	42
A-SICI60	57.8 ± 19.6	0.287	0.446	0.511	0.676	0.394	0.565	69	59	49
A-SICI70	30.6 ± 18.2	0.371	0.541	0.373	0.543	0.513	0.678	52	55	40
A-SICI80	22.1 ± 11.1	0.329	0.496	0.424	0.595	0.541	0.702	32	29	24
A-SICI slope	199.4 ± 49.4	0.173	0.295	0.511	0.676	0.449	0.620	179	152	120
peak A-SICI	17.4 ± 9.8	0.423	0.595	0.510	0.675	0.576	0.731	28	28	20
T-SICI50	2.3 ± 4.7	0.414	0.586	0.529	0.700	0.582	0.736	15	9	9
T-SICI60	10.4 ± 13.0 5.7 (8.4)^c^	0.924	0.961	0.883	0.938	0.925	0.961	10	12	10
T-SICI70	20.4 ± 15.9	0.923	0.96	0.615	0.761	0.651	0.789	14	27	28
T-SICI80	23.0 ± 14.6	0.809	0.895	0.608	0.756	0.858	0.923	22	25	16
T-SICI slope	56.1 ± 39.1	0.88	0.936	0.827	0.905	0.870	0.930	43	42	40
peak T-SICI	28.8 ± 17.7	0.871	0.931	0.816	0.899	0.893	0.944	20	20	16

Mean ± SD and coefficient of repeatability are expressed as % MSO for RMT_0.2mV_ and TS_1mV_, % test MEP for A-SICI and % RMT_0.2mV_ for T-SICI parameters; intraclass correlation coefficient (ICC) is a dimensionless measure. SD – standard deviation; SDC - smallest detectable change.

a Averaged across four sessions.

b Two subsequent measurements made on the same day averaged and used to calculate interday reliability.

c Median (interquartile range).

d Single measures.

e Averaged measures.

In approximately two thirds of the recordings, peak inhibition was observed at CS 80% RMT_0.2mV_ (28 and 32 out of 48 recordings for A-SICI and T-SICI, respectively) and only rarely at CS 60% RMT_0.2mV_ (in 3 out 48 recordings with each technique). However, the agreement between the CS intensity at which peak SICI was observed was poor both within and between the experimental days with either of the methods (Cohen's kappa −0.105-0.360, p > 0.11).

There was no significant difference in average TMS measurements between sexes (Student t-test, p > 0.5; Mann-Whitney *U* test, p > 0.4) or TA and AT protocol sequences (Student t-test, p > 0.1; Mann-Whitney *U* test, p > 0.2).

### Comparability of the two techniques

**Fig. 3:**
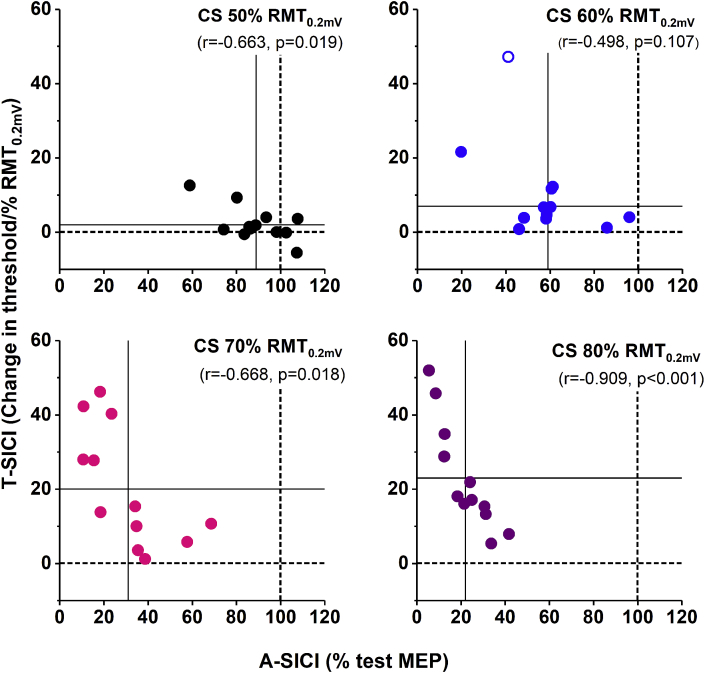
**Comparability of A-SICI and T-SICI at individual conditioning stimulus intensities.** The scatter plots demonstrate the relationship between SICI obtained by conventional technique (x axis) and threshold-tracking (y axis). Circles indicate SICI averaged across four sessions for each subject, open circle indicates one potential outlier. Different colours indicate CS intensities; dotted lines represent the test conditions (100% test MEP for A-SICI, 0% RMT_0.2mV_ for T-SICI), solid lines – group means. Pearson's correlation coefficients were calculated for average SICI. SICI conditions at CS intensity of 50, 70, and 80% RMT_0.2mV_ showed significant strong negative linear correlations. At CS intensity of 60% RMT_0.2mV_, the correlation improved when an outlier (open circle) was removed from the analysis (r = −0.581, p = 0.061). (For interpretation of the references to colour in this figure legend, the reader is referred to the Web version of this article.)

**Fig. 4:**
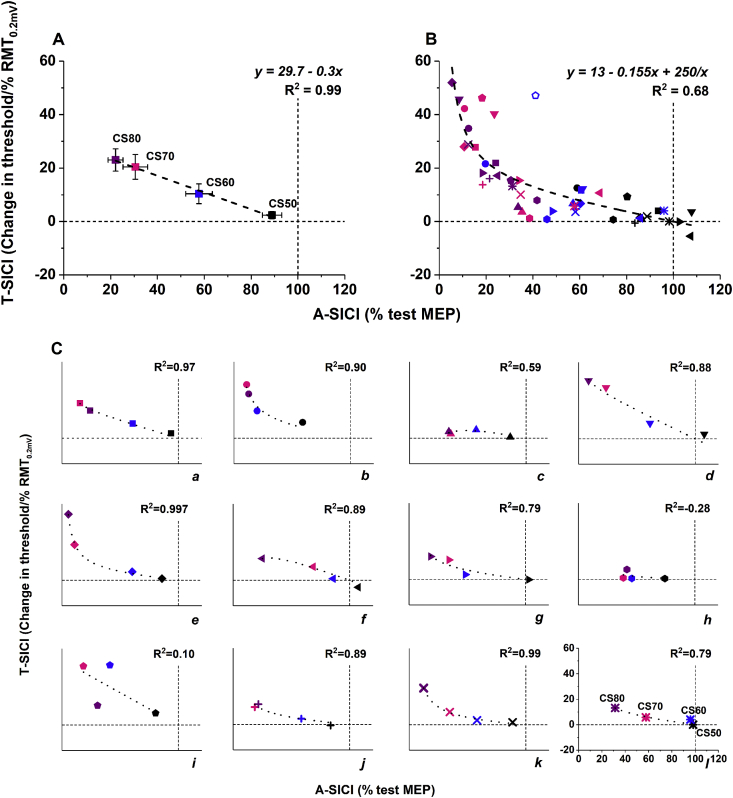
**Relationship between A-SICI and T-SICI recruitment curves.** Scatter plots demonstrate the relationship between mean SICI recruitment curves obtained by conventional technique (x axis) and threshold-tracking (y axis). Dotted lines indicate control conditions (100% test MEP for A-SICI, 0% RMT_0.2mV_ for T-SICI). A) A strong linear relationship between the mean group SICI obtained by the two techniques. Group means were obtained by averaging SICI at matching conditioning stimulus levels (indicated by colours and labels: black/CS50 - CS 50% RMT_0.2mV_; blue/CS60 - CS 60% RMT_0.2mV_; pink/CS70 - CS 70% RMT_0.2mV_; purple/CS80 - CS 80% RMT_0.2mV_; dashed line – linear fit, error bars – standard error of the mean). B) A non-linear relationship between individual SICI means averaged across four sessions for each subject. Individual SICI recruitment curves from C) were superimposed (symbols represent different subjects and correspond to symbols in C); colours – conditioning stimulus levels as in A); dashed line - best fitting curve with two parameters satisfying the boundary conditions y = 0 when x = 100 and y = infinity when x = 0 [y = a(100-x)+b(100-x)/x]). Fit further improved when an outlier (open symbol) was removed (y = 9.94–0.127x+276/x, R^2^ = 0.78). C) The relationship between A-SICI and T-SICI varied among individuals over the same range of conditioning stimulus levels. Data points indicate SICI averaged across four sessions (symbols correspond to subject's symbol in B); colours and labels indicate conditioning stimulus levels as in A) and B); thick dotted line – fitted curve y = a(100-x)+b(100-x)/x). In almost half of the subjects, the relationship was near linear (e.g., a, d, l), while a ‘floor effect’ with conventional method was observed in others (e.g., b, e). In some subjects, no apparent correlation between A-SICI and T-SICI recruitment curves was seen (e.g., i). (For interpretation of the references to colour in this figure legend, the reader is referred to the Web version of this article.)

To assess the relationship between A-SICI and T-SICI, data was averaged across four sessions for each individual and correlated [38]. Significant negative linear correlations between the two techniques were found at peak SICI and SICI slope (Pearson's r = −0.847, p < 0.001 and r = −0.665, p = 0.018, respectively) as well as all but 60% RMT_0.2mV_ CS intensities (Fig. 3). Although on the group level the relationship between mean SICI recruitment curves obtained by two different techniques was linear (Fig. 4, A), a considerable inter-individual variability was observed (Fig. 4, C). Across all subjects, a strong non-linear relationship between A-SICI and T-SICI slopes was seen (Fig. 4, B), which can be explained by the ‘floor’ effect observed in the conventional method at strong inhibition levels.

Both conditioning and test stimulus intensities are important in determining the magnitude of SICI [[39], [40], [41]]. While in the conventional technique stimulation intensities are constant, they are continuously adjusted in threshold-tracking. Raw CS intensities (in % MSO) used in this experiment did not differ between the techniques at any of the SICI conditions (paired-sample *t*-test, p > 0.1), while test stimulus intensities reached in T-SICI at CS 50–60% RMT_0.2mV_ were significantly lower than the TS_1mV_ used for A-SICI (absolute difference of 7 ± 4.7% and 3 ± 2.9% MSO, respectively; paired-sample *t*-test, p < 0.05). Thresholds at CS 70–80% RMT_0.2mV_ were 2 ± 5.5% and 3 ± 9.4% MSO higher than TS_1mV_ intensity used in the conventional technique, but this was not significant (paired-sample *t*-test, p > 0.2).

### Reproducibility of TMS parameters

Several methods have been proposed to quantify the reliability of measurements. Relative reliability, or reproducibility, indicates the degree to which subjects maintain their position within a group over repeated measurements [42,43]. This is quantified using intraclass correlation coefficient (ICC) [34,43]. Reproducibility of a test has important implications in interventional studies. Fleiss suggested that using unreliable outcome measure increases the sample size required to detect a significant treatment effect by 1/ICC [34] and the effect of reproducibility on statistical power has been demonstrated in a recent study [44].

**Fig. 5:**
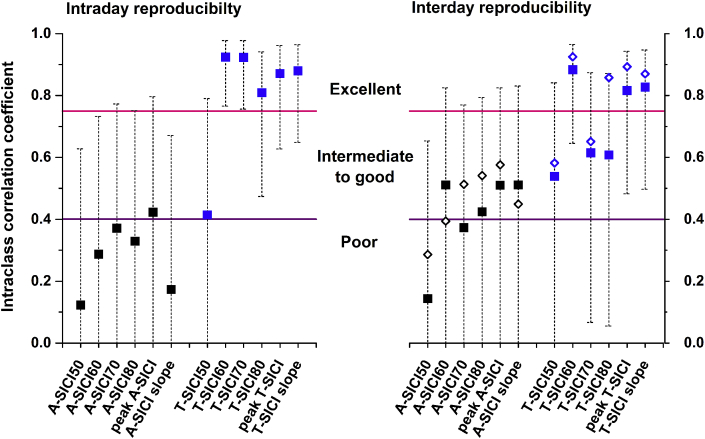
**Reproducibility of SICI.** Recordings from Day 1 (runs 1 and 2) were used to assess the intraday reproducibility of SICI estimates and results of the first session on Days 1 and 2 (i.e., runs 1 and 3) – for interday reproducibility (filled squares). A-SICI had poor intra- and poor-to-adequate interday reproducibility, while T-SICI showed adequate-to-excellent intra- and interday reproducibility. Single measures [ICC (2,1)] model data is presented. Error bars represent 95% confidence intervals for intraclass correlation coefficients. Averaging two SICI estimates obtained on the same day (i.e. runs 1 and 2 on Day 1, runs 3 and 4 on Day 2) did not improve the interday reproducibility considerably (open diamonds), except maybe for T-SICI80. The ICC of T-SICI60 dropped for intraday from 0.92 to 0.78 and interday from 0.88 to 0.5 when the measurement of one outlier with strong inhibition (more than 3 IQRs outside the boxplot) was removed from the analysis, but this did not affect the measurement error (see Supplementary Table 1). This illustrates the counter-intuitive aspects of the ICC that it increases if the heterogeneity of a sample increases even if the measurement error stays unaltered.

Recordings from Day 1 (runs 1 and 2) were used to assess the intraday reproducibility, and recordings from the first session of the experimental day (runs 1 and 3) - interday reproducibility (Fig. 5). Interday reliability was also assessed using averaged measurements obtained subsequently on the same experimental day (Table 1).

RMT_0.2mV_ showed high reliability comparable to previously reported [8,29,44]. Overall, SICI parameters obtained by threshold-tracking showed adequate-to-excellent reproducibility, while most conventional measurements tended to have poorer reproducibility (Fig. 5). T-SICI60, peak T-SICI and T-SICI slope were the most reliable measurements both within and between the experimental days. The interday reproducibility of some SICI parameters (e.g. A-SICI70, T-SICI80) improved by averaging values from same-day recordings. Although a trend towards better reproducibility was seen with T-SICI, it did not prove to be statistically significant due to broad and overlapping 95% confidence intervals of ICCs for both techniques (Fig. 5).

### Repeatability of TMS measurements

ICC is a dimensionless estimate that indicates how well a test can differentiate between the rank order of individuals with test repetition (the individuals with smallest or largest SICI value remain at the bottom and top of a cohort) [8,43], but it does not provide information on the absolute differences between repeated measurements [33]. However, in clinical practice absolute reliability, i.e. the agreement between repeated measurements (a given amount of SICI) in an individual, is more important for determining the suitability of a diagnostic test for individual decision making. This can be assessed using Bland–Altman plots [35] or coefficient of repeatability (CR) - a value below which the differences of future measurements within a subject will lie with 95% probability [37]. CR is derived from standard error of measurement (Supplementary Table 1) and is equivalent to the smallest detectable change (SDC), which indicates a true change in test score beyond the measurement noise [8,45].

**Fig. 6:**
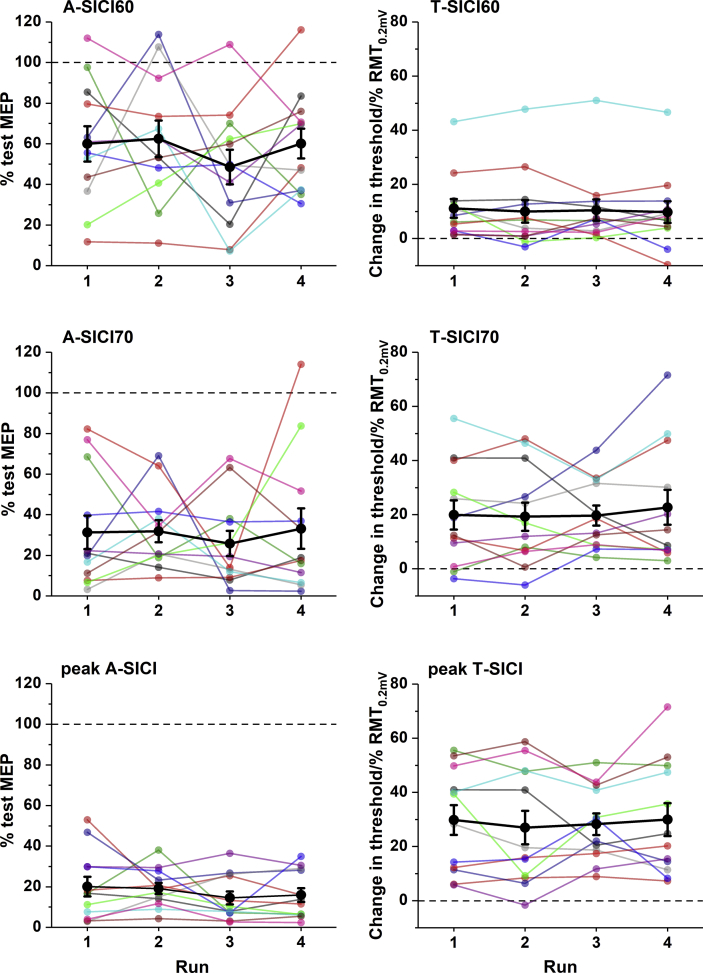
**Variability of SICI over time.** There were no significant differences between the sessions in mean group SICI. Although some subjects had relatively stable measurements over time, most individuals showed fluctuations which could differ by a factor of more than 10 times from the initial measurement. Large black circles indicate group means, error bars - standard error of the mean, small coloured circles – raw values of each subject.

Although in our study there were no significant differences in group means of repeated measurements, fluctuations in some parameters over time in individual subjects showed up to 10-fold differences from the initial measurements (Fig. 6). Overall, agreement between TMS measurements was poor (CRs summarized in Table 1). It was similar within and between the days (except for RMT_0.2mV_ and T-SICI70, for which the intraday repeatability was twice better than interday) and did not improve considerably if two same-day estimates were averaged.

**Fig. 7:**
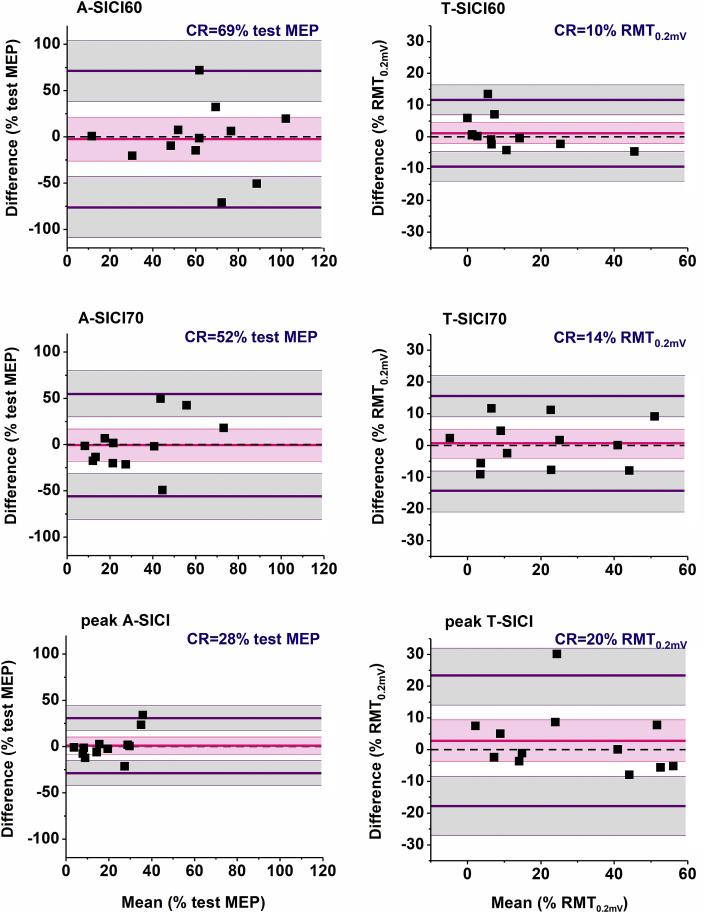
**Intraday repeatability of SICI measurements.** None of the SICI measurements showed a significant bias between the recording sessions within the same experimental day (runs 1 and 2). However, broad 95% limits of agreement and large coefficients of repeatability indicate the considerable variability of these parameters within subjects. For example, if initial measurement of A-SICI70 was 50% test MEP, a repeat measurement between 0 and 102% test MEP would not be considered a significant change. Similarly, if initial T-SICI70 was 20% RMT_0.2mV_, a repeat measurement between 6 and 34% RMT_0.2mV_ would reflect a measurement noise, not a true change. Difference between sessions was calculated as (Run 1 - Run 2). Dots represent data of individual subjects, bold pink line indicates mean difference (bias), bold purple lines - upper and lower 95% limits of agreement. Shaded areas represent 95% confidence intervals for bias (light pink) and 95% limits of agreement (grey). Black dotted line - line of identity. CR – coefficient of repeatability. (For interpretation of the references to colour in this figure legend, the reader is referred to the Web version of this article.)

Bland–Altman plots showed no significant bias between repeated measurements for any of the SICI parameters, suggesting there was no systematic error between the runs. However, the limits of agreement were broad for all SICI conditions obtained with both techniques (Fig. 7). Although these findings could partially be explained by a small sample size [33], they also point towards a substantial variability of SICI measurements, irrespective of the recording technique.

Repeatability is also of importance for sample size calculation in interventional studies. Our findings indicate that the use of T-SICI may reduce a required sample size compared to conventional technique (supplementary material).

### Protocol duration

Threshold-tracking protocol for SICI was significantly shorter than the conventional amplitude method (3.8 (IQR 1.2) vs 5.8 (IQR 0.3) minutes; Wilcoxon signed rank test, p < 0.001). The estimation of RMT_0.2mV_ by threshold-tracking lasted less than 90 s on all occasions and required a median of 10 (IQR 2) stimuli. The TS_1mV_ estimation for A-SICI was of similar duration (11 (IQR 4) stimuli).

## Discussion

The principle finding of this study is that in a direct head-to-head comparison, SICI obtained by the two techniques showed good correlation without group difference in any of the parameters between recording sessions. T-SICI was quicker to perform and tended to have better reproducibility compared to conventional A-SICI, especially if recorded on the same day. However, both techniques had poor agreement in repeated measurements of individual subjects.

### Reliability of SICI measurements

Previously reported reproducibility of conventional A-SICI varied greatly between studies with ICCs ranging from 0.23 to 0.91 [8,[26], [27], [28], [29]] and our measurements fall into this range. ICC values should not be interpreted in isolation, and factors such as model, precision, and heterogeneity of the sample must always be taken into consideration [8,37,44]. In most of the previous studies, the ICC model was either not specified or a model with average measures [ICC(2,k), ICC(3,k)] was used, which is known to increase the ICC value but would be inappropriate in those circumstances where a single measurement is used as an outcome [43,44]. The precision of the ICC estimates (i.e. 95%CIs) was rarely presented. Most importantly, sample heterogeneity highly impacts the ICC and must be considered when different studies are compared [8,37,46]. Using an average of repeated measurements is known to improve the reliability of a test [34], but in our study this resulted only in a marginal improvement (Fig. 5).

In our study, ICC estimates for T-SICI were higher than those of A-SICI, especially when measured on the same day. However, since in our small sample the 95%CIs were large and overlapping, the difference did not reach statistical significance. Our sample size was adequate to detect ICCs of >0.9 with 95%CIs of 0.2, but about 100–300 subjects more would be required to achieve the same precision when ICC falls between 0.7 and 0.3 (the lower the ICC, the larger the sample size needed) [47]. This could mean that T-SICI is potentially superior to conventional method at detecting inter-individual differences and may be more suitable for discriminative purposes (e.g., disease staging) [45]. However, reproducibility estimates of SICI in groups of healthy individuals cannot be extrapolated to pathological conditions, especially if patients are a very homogeneous group (i.e., all have markedly reduced/absent SICI) and therefore need to be evaluated independently.

The relationship between SICI and CS intensity is non-linear and varies between individuals [5,32], therefore using a single CS intensity may contribute to the variability of the outcome. With threshold-tracking, parameters obtained from SICI recruitment curve (T-SICI slope and peak T-SICI) showed better reproducibility (Fig. 5) and smaller relative measurement error (Supplementary Table 1) than individual SICI conditions between the experimental days. Thus using a range of CS intensities may provide a more stable T-SICI measure over time.

In clinical practice, high measures of absolute reliability are essential for the diagnostic value of a test in an individual (e.g. assessment of treatment response). Only a few previous studies explored this reliability of conventional SICI in healthy individuals and they reported CRs greatly varying from 17% to 147% test MEP [8,28,29]. In our study, the CRs (aka smallest detectable changes) were high for both techniques, irrespective of whether the measurements were taken on the same day or at least one week later. Calculation of CR is closely related to the Bland–Altman's 95% limits of agreement which will be wide if obtained from a small sample size [33,37]. Although small sample size could partially explain poor agreement of SICI measurements in this study, overall the data is suggestive of a substantial biological variability that is currently unexplained.

### Comparability of A-SICI and T-SICI: are we assessing the same neuron pools?

The faster acquisition speed and potential for higher reproducibility make T-SICI more appealing than A-SICI. But can these two techniques be used interchangeably?

Some phenomena were observed independently with both techniques, such as the ISIs for peak inhibition [[2], [3], [4],^30^], the effect of CS intensity [2,40] and voluntary activation on SICI [3,48], and overlap with short-interval intracortical facilitation [3,7,39,49]. However, a head-to-head comparison of the two techniques has never been done before.

In our study, subjects who had strong inhibition measured by conventional method also showed strong inhibition when threshold-tracking was used. This was true for most individual SICI conditions, with SICI at CS 80% RMT_0.2mV_ and peak SICI showing the strongest correlation between the techniques. On a group level the correlation between mean A-SICI and T-SICI was strongly linear across the studied CS intensity range. However, non-linear correlations with notably different slopes were evident in some individuals which could reflect the ‘floor’ effect seen with conventional technique or suggest that in some subjects different subsets of neuronal pools were engaged.

**Fig. 8:**
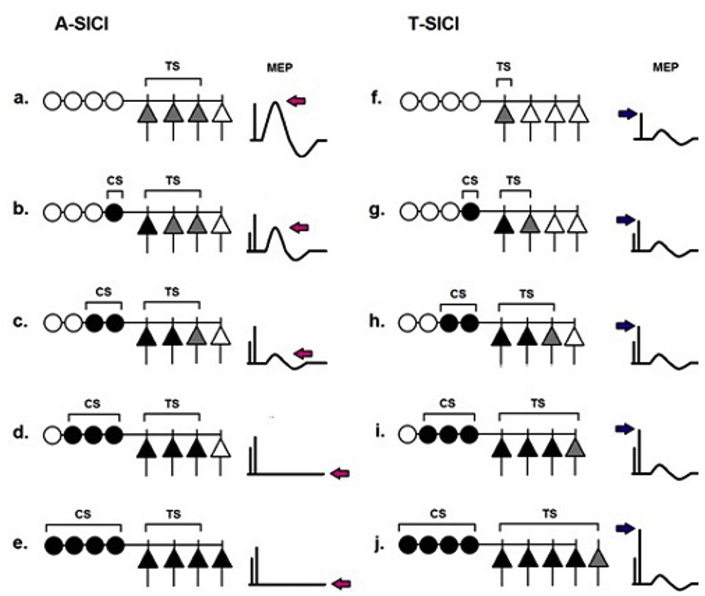
**Schematic illustration of hypothetical neuronal pools assessed by conventional (A-SICI) and threshold-tracking (T-SICI) techniques.** This diagram is based on an assumption that the size of the neuron pool under investigation is defined by the stimulation intensity. Triangles represent upper (cortical) motoneuron pool, circles – inhibitory interneurons projecting onto motoneurons. In A-SICI, the size of the motoneuron pool (represented in a by 3 of 4 neurons; grey triangles) that will be tested and will generate an unconditioned motor response is pre-determined by the test stimulus (TS) intensity. As the intensity of the conditioning stimulus increases (b to d), inhibitory (GABA-ergic) interneurons are progressively recruited (black circles), exerting increasingly stronger inhibitory effect on the upper motoneuron pool (black triangles). As a result the conditioned MEP amplitude decreases (b, c) and is eventually abolished (d). Although even more inhibitory interneurons might be recruited by stronger conditioning stimuli (e), this cannot be further quantified (as the inhibited neurons are not activated by the test stimulus) thus producing a ‘floor effect’. By contrast, in T-SICI, test stimulus intensity is adjusted to counteract the effect of the inhibitory interneurons so that a small response (represented by a single grey neuron) is always obtained (g to j). Although potentially different subsets of motoneurons are assessed at different conditioning stimulus levels, this allows the inhibitory potential of GABA-ergic interneuron pool to be fully evaluated. Arrows indicate change in MEP amplitude (pink) and test stimulus intensity (blue). (For interpretation of the references to colour in this figure legend, the reader is referred to the Web version of this article.)

Stimulation intensity is an important consideration when comparing the two techniques. Whereas the TS_1mV_ intensity used in the conventional A-SICI was optimal for eliciting maximum inhibition [41] and was constant for all SICI conditions, stimulation intensities in T-SICI varied depending on the CS intensity. It is likely that different subsets of upper motoneurons are engaged at different test stimulus intensities (Fig. 8). Other factors, such as slope of stimulus-response function, overlap with short-interval intracortical facilitation, interactions at the spinal level may be important for the comparison of the two techniques and are yet to be systematically investigated. Nevertheless, the close relationship between A-SICI and T-SICI across a range of conditions suggests that the neurons explored by the two techniques have much in common. Future pharmacological interventions may provide further insight into the similarities and differences of the neuronal pathways interrogated by the two techniques.

In this study, the exclusion rate due to coil overheating was relatively high and biased towards subjects with high RMT_0.2mV_, posing a potential limitation to the results. CS intensities for A-SICI were set based on RMT_0.2mV_ which, according to limited reports, is equal to about 109% RMT_0.05mV_ [50,51]. This should be kept in mind when comparing our results to the studies where RMT_0.05mV_ was used. It is also important to note that our data is limited to a single interstimulus interval and used stimulation parameters. Future experiments should address whether the relationship between A-SICI and T-SICI as well as their reliability is similar across the whole range of ISIs.

A-SICI might be a more appropriate method if one is interested in investigating effects of an intervention on a particular motoneuron subset. However, if a single SICI condition is used and baseline inhibition is strong, the ‘floor effect’ might prevent fully quantifying SICI-enhancing effects. It could be avoided by adjusting CS intensity to produce 50% inhibition at baseline [52], but this would result in varying CS intensities in relation to individual motor thresholds and may introduce bias. Recording SICI recruitment curve thus would be favourable. T-SICI allows the inhibitory potential to be fully evaluated and might be better at detecting inter-individual differences within a group as well as outliers. Threshold-tracking is potentially quicker and could be advantageous where time constraints are important. Based on our data, smaller sample sizes may be sufficient to detect SICI-enhancing effect of a drug of a similar magnitude to that seen with benzodiazepines [52,53] in a cross-over experiment if threshold-tracking was used (Supplementary Table 2). However, the two techniques have not been compared in interventional studies so far and it is yet to be determined whether their sensitivity to intervention is similar. A potential limitation of T-SICI is a ‘ceiling effect’ in subjects with high resting motor thresholds (>60–65% MSO) as the magnetic stimulator will run out of power to demonstrate full inhibition which would require MSO of >100%.

In summary, the recently developed T-SICI technique shows great promise for improved speed and reproducibility of data acquisition over the conventional method and therefore deserves further investigation.

## Funding

This research was funded by the Medical Research Council (grant ref. MR/K015222/1). The funding source did not have any involvement in study design, data collection, analysis, and interpretation, in the writing of this report, and in the decision to submit the article for publication.

## Declaration/conflicts of interest

Hugh Bostock receives from UCL a share of the royalties for sales of his Qtrac software used in this study. Other authors have no conflicts of interest to declare.
