# Supplementary material for: Short-interval intracortical inhibition: Comparison between conventional and threshold-tracking techniques
Source: Brain Stimul. 2018 Jul-Aug;11(4):806–17. doi: 10.1016/j.brs.2018.03.002 (PMC6028741; doi:10.1016/j.brs.2018.03.002)
Supplement: Supplementary material [file mmc1.docx]

**Supplementary material**

| **Supplementary table 1. SICI measurement error** | | | | | |
| --- | --- | --- | --- | --- | --- |
| **Parameter** | **Intraday** | |  | **Interday** | |
|  | **Absolute SEM_eas_** | **Relative SEM_eas_** |  | **Absolute SEM_eas_** | **Relative SEM_eas_** |
| A-SICI50 | 21 | 0.24 |  | 23 | 0.27 |
| A-SICI60 | 25 | 0.41 |  | 21 | 0.38 |
| A-SICI70 | 19 | 0.60 |  | 20 | 0.69 |
| A-SICI80 | 12 | 0.50 |  | 10 | 0.50 |
| A-SICI slope | 65 | 0.32 |  | 55 | 0.29 |
| peak A-SICI | 10 | 0.52 |  | 10 | 0.58 |
| T-SICI50 | 5 | 3.54 |  | 3 | 1.22 |
| T-SICI60 | 4 (4)* | 0.34 (0.54)* |  | 4 (4)* | 0.40 (0.53)* |
| T-SICI70 | 5 | 0.25 |  | 10 | 0.49 |
| T-SICI80 | 8 | 0.35 |  | 9 | 0.38 |
| T-SICI slope | 15 | 0.28 |  | 15 | 0.26 |
| peak T-SICI | 7 | 0.25 |  | 7 | 0.24 |
| *Standard error of measurement (SEM_eas_) indicates the accuracy of repeated measurements (i.e. the size of measurement error). Absolute SEM_eas_ is expressed on the same scale as the measurement (% test MEP for A-SICI, % RMT for T-SICI), while relative SEM_eas_ reflects the size of the measurement error as a proportion of the group mean (absolute SEM_eas_/group mean), thus allowing to compare the relative noisiness of different measurements [1]; * numbers in the brackets indicate SEM_eas_ calculated without an outlier.* | | | | | |

Sample size calculation for interventional studies

The reliability data from our study can be used for interventional study planning. For example, we are planning a cross-over study in which we will use an intervention that from previous work is known to increase conventional A-SICI at CS 70% RMT by 10-20% test MEP. From our study, we know that the intraday within-subject variability in repeated measurements of A-SICI70 due to measurement error is 19% test MEP. Therefore, for a cross-over interventional study to have a power of 80% to detect a change in A-SICI70 of 10-20% test MEP on a two-sided 0.05 significance level, between 59 and 17 subjects would be required. With threshold-tracking (T-SICI70), a smaller sample (between 46 and 13 subjects) may be sufficient to detect a comparable effect with the same power and significance level (supplementary table 2).

| **Supplementary table 2. Sample size estimation for a two-treatment cross-over design experiment (pre- and post-intervention assessments carried out on the same day)** | | | | | | | |
| --- | --- | --- | --- | --- | --- | --- | --- |
| **A-SICI60** | | |  | **T-SICI60** | | | |
| **SD_WS_**  (%test MEP) | **MDD**  (% test MEP) | **n** |  | **SD_WS_**  (% RMT) | | **MDD**  (% RMT) | **n** |
| 25 | 10 | *101* |  | 4 | | 3 | *30* |
|  | 20 | *27* |  |  |  | 6 | *10* |
|  | 30 | *13* |  |  |  | 9 | *6* |
|  | 40 | *9* |  |  |  | 12 | *5* |
|  | 50 | *7* |  |  |  | 15 | *4* |
|  | 69* | *5* |  |  |  | 10* | *5* |
| **A-SICI70** | | |  | **T-SICI70** | | | |
| **SD_WS_**  (%test MEP) | **MDD**  (% test MEP) | **n** |  | **SD_WS_**  (% RMT) | **MDD**  (% RMT) | | **n** |
| 19 | 10 | *59* |  | 5 | 3 | | *46* |
|  | 20 | *17* |  |  | 6 | | *13* |
|  | 30 | *9* |  |  | 9 | | *7* |
|  | 40 | *6* |  |  | 12 | | *5* |
|  | 50 | *5* |  |  | 15 | | *5* |
|  | 52* | *5* |  |  | 14* | | *5* |
| **peak A-SICI** | | |  | **peak T-SICI** | | | |
| **SD_WS_**  (%test MEP) | **MDD**  (% test MEP) | **n** |  | **SD_WS_**  (% RMT) | **MDD**  (% RMT) | | **n** |
| 10 | 10 | *18* |  | 7 | 3 | | *88* |
|  | 20 | *7* |  |  | 6 | | *24* |
|  | 30 | *5* |  |  | 9 | | *12* |
|  | 40 | *4* |  |  | 12 | | *8* |
|  | 50 | *3* |  |  | 15 | | *6* |
|  | 28* | *5* |  |  | 20* | | *5* |
| *Sample size (n) was calculated at 0.05 significance level (2-sided) and 0.8 power using within-subject standard deviation (SD_WS_) and minimal detectable difference in means (MDD) as predefined parameters [2]. SD_WS_ reflects the within-subject variation due to measurement error, the presented values were observed in our study. The MDD values represent the expected absolute change (increase or decrease) in SICI and * denotes smallest detectable change observed in our study. MDDs for T-SICI were matched to A-SICI (except the ones marked with *), obtaining the values from the linear relationship T-SICI = 29.7-0.3 x A-SICI (Figure 4, A in the manuscript). Grey areas indicate the change in SICI which is higher than the group means observed in our sample. Enhancing effects of this magnitude would not be demonstrable with conventional technique (A-SICI) due to ‘floor‘ effect, while such limitation does not apply to T-SICI or SICI-decreasing effects with either technique.* | | | | | | | |

| **Supplementary table 3. Sample size estimation for a two-treatment cross-over design experiment (pre- and post-intervention assessments carried out at least one week apart)** | | | | | | | |
| --- | --- | --- | --- | --- | --- | --- | --- |
| **A-SICI60** | | |  | **T-SICI60** | | | |
| **SD_WS_**  (%test MEP) | **MDD**  (% test MEP) | **n** |  | **SD_WS_**  (% RMT) | | **MDD**  (% RMT) | **n** |
| 21 | 10 | *72* |  | 4 | | 3 | *30* |
|  | 20 | *20* |  |  |  | 6 | *10* |
|  | 30 | *10* |  |  |  | 9 | *6* |
|  | 40 | *7* |  |  |  | 12* | *5* |
|  | 50 | *5* |  |  |  | 15 | *4* |
|  | 59* | *5* |  |  |  |  |  |
| **A-SICI70** | | |  | **T-SICI70** | | | |
| **SD_WS_**  (%test MEP) | **MDD**  (% test MEP) | **n** |  | **SD_WS_**  (% RMT) | **MDD**  (% RMT) | | **n** |
| 20 | 10 | *65* |  | 10 | 3 | | *177* |
|  | 20 | *18* |  |  | 6 | | *46* |
|  | 30 | *10* |  |  | 9 | | *22* |
|  | 40 | *7* |  |  | 12 | | *13* |
|  | 50 | *5* |  |  | 15 | | *10* |
|  | 55* | *5* |  |  | 27* | | *5* |
| **peak A-SICI** | | |  | **peak T-SICI** | | | |
| **SD_WS_**  (%test MEP) | **MDD**  (% test MEP) | **n** |  | **SD_WS_**  (% RMT) | **MDD**  (% RMT) | | **n** |
| 10 | 10 | *18* |  | 7 | 3 | | *88* |
|  | 20 | *7* |  |  | 6 | | *24* |
|  | 30 | *5* |  |  | 9 | | *12* |
|  | 40 | *4* |  |  | 12 | | *8* |
|  | 50 | *3* |  |  | 15 | | *6* |
|  | 28* | *5* |  |  | 20* | | *5* |
| *Sample size (n) was calculated at 0.05 significance level (2-sided) and 0.8 power using within-subject standard deviation (SD_WS_) and minimal detectable difference in means (MDD) as predefined parameters [2]. SD_WS_ reflects the within-subject variation due to measurement error, the presented values were observed in our study. The MDD values represent the expected absolute change (increase or decrease) in SICI and * denotes smallest detectable change observed in our study. MDDs for T-SICI were matched to A-SICI (except the ones marked with *), obtaining the values from the linear relationship T-SICI = 29.7-0.3 x A-SICI (Figure 4, A in the manuscript). Grey areas indicate the change in SICI which is higher than the group means observed in our sample. Enhancing effects of this magnitude would not be demonstrable with conventional technique (A-SICI) due to ‘floor‘ effect, while such limitation does not apply to T-SICI or SICI-decreasing effects with either technique.* | | | | | | | |

Reference

[1] Schambra HM, Ogden RT, Martinez-Hernandez IE, Lin X, Chang YB, Rahman A, et al. The reliability of repeated TMS measures in older adults and in patients with subacute and chronic stroke. Front Cell Neurosci 2015;9:335.

[2] Schoenfeld D. Statistical considerations for clinical trials and scientific experiments [updated 07 January 2015; accessed 03 October 2017]. Available from: <http://hedwig.mgh.harvard.edu/sample_size/size.html>.
